# Oncosurgical Management of Liver Limited Stage IV Colorectal Cancer: Preliminary Data and Protocol for a Randomized Controlled Trial

**DOI:** 10.2196/resprot.9453

**Published:** 2018-05-09

**Authors:** Paul Sutton, Dale Vimalachandran, Graeme Poston, Stephen Fenwick, Hassan Malik

**Affiliations:** ^1^ Aintree University Hospital Liverpool United Kingdom; ^2^ Countess of Chester Hospital Chester United Kingdom

**Keywords:** colorectal cancer, liver metastases

## Abstract

**Background:**

Colorectal cancer is the fourth commonest cancer and second commonest cause of cancer-related death in the United Kingdom. Almost 15% of patients have metastases on presentation. An increasing number of surgical strategies and better neoadjuvant treatment options are responsible for more patients undergoing resection of liver metastases, with prolonged survival in a select group of patients who present with synchronous disease. It is clear that the optimal strategy for the management of these patients remains unclear, and there is certainly a complete absence of Level 1 evidence in the literature.

**Objective:**

The objective of this study is to undertake preliminary work and devise an outline trial protocol to inform the future development of clinical studies to investigate the management of patients with liver limited stage IV colorectal cancer.

**Methods:**

We have undertaken some preliminary work and begun the process of designing a randomized controlled trial and present a draft trial protocol here.

**Results:**

This study is at the protocol development stage only, and as such no results are available. There is no funding in place for this study, and no anticipated start date.

**Conclusions:**

We have presented preliminary work and an outline trial protocol which we anticipate will inform the future development of clinical studies to investigate the management of patients with liver limited stage IV colorectal cancer. We do not believe that the trial we have designed will answer the most significant clinical questions, nor that it is feasible to be delivered within the United Kingdom’s National Health Service at this current time.

## Introduction

Colorectal cancer is the fourth commonest cancer and second commonest cause of cancer-related death in the United Kingdom [1]. Almost 15% of patients have metastases on presentation [2]. An increasing number of surgical strategies and better neoadjuvant treatment are bringing more patients to resection of liver metastases with prolonged survival in a select group of patients who present with synchronous disease [3].

It is clear that the optimal strategy for the management of these patients remains unclear, and there is certainly a complete absence of level 1 evidence in the literature. In brief, the multidisciplinary team is faced with three potential surgical strategies: resection of the primary tumor, followed by resection of the liver metastases; resection of the liver metastases, followed by resection of the primary tumor; or simultaneous (synchronous) resection of disease at both sites in a combined operation.

A recent systematic review identified 18 publications comparing simultaneous and staged (classic or reverse) procedures that had more than 10 patients in each group [4]. No significant difference in mortality was found between these surgical strategies; however, statistically significant differences were found in duration of operation, blood loss, length of hospital stay, and morbidity. Four publications favored the simultaneous approach in terms of cumulative duration of procedure, whereas in two there was no difference. Four publications favored the simultaneous approach as regards blood loss, and four found no significant difference. All studies that compared length of hospital stay favored the simultaneous approach. Most importantly, three publications favored the simultaneous approach in terms of morbidity [5-7], whereas 7 others did not detect any difference [8-14].

The authors of the review paper conclude that no one strategy is inferior to the others and that all of them should be considered in patients presenting with synchronous colorectal liver metastases. The clear contraindication to this approach would be those patients with a symptomatic primary tumor, for example, those that are bleeding and requiring transfusion, perforated tumors, and those obstructing or with an imminent threat of the same. The biggest question arising from these studies is whether or not it is possible to identify subgroups of patients who would benefit from one particular strategy over another, particularly if considering that those patients presenting with synchronous disease may represent a distinct biological subtype [15]. Unfortunately the evidence is lacking, with a complete absence of randomized data and multiple inadequacies in existing cohort studies. The most appropriate course of management for these patients is currently determined on an individual basis by specialty multidisciplinary teams [16]. There is, therefore, a clear need for high-quality clinical evidence to empower clinicians to adequately plan surgical management.

The prospective data available suggest it may be possible to reduce surgical morbidity with a simultaneous approach. In addition to this, there is the possibility of reducing the negative impact on quality of life (QoL) and time to resumption of normal activities by performing a single (albeit more complex) operation compared with two operations. A single operation may also confer an earlier commencement of adjuvant chemotherapy and the potential for better systemic control. Finally, the National Health Service (NHS) reference costs for surgical procedures suggest that cost is predominantly a feature of operating time, critical care admission, and length of stay. A single operation may generate a lower combined figure for all of these factors resulting in cost savings.

In light of these factors, we have undertaken some preliminary work and begun the process of designing a randomized controlled trial (RCT) and present a draft trial protocol here. We do not believe, however, that the trial we have designed will answer the most significant clinical questions, nor that it is feasible to be delivered within the UK’s NHS at this current time.

## Methods

### Objectives of the Trial

#### Primary Objective

The primary objective was to establish the feasibility of conducting an RCT comparing simultaneous and staged resections in liver limited metastatic colorectal cancer.

#### Secondary Objectives

Secondary objectives include investigating for differences between simultaneous and staged resections with respect to surgical morbidity, disease free and overall survival, the proportion of patients in which R0 resection at both sites is achieved, and the time to commencement of adjuvant systemic therapy (if planned); cost to the NHS; QoL and return to work; and patient-reported outcome measures (PROMs; to be developed within this study).

### End Points

Our primary end point is willingness of multidisciplinary teams (MDTs) to randomize patients. In addition to this, the following secondary end points will also be considered:

- Total number of (severe) surgical complications (Clavien-Dindo grade 1-5)
- Disease-free survival
- Overall survival
- Achievement of R0 resection at both sites
- Completion of planned adjuvant therapy
- Time to commencement of adjuvant chemotherapy (if planned)
- Total cost of operative treatment
- QoL
- Return to work

### Patient Selection Criteria

The inclusion criteria for this study is shown in Textbox 1.

### Trial Design

The trial is a two-armed phase II randomized (1:1) controlled difference trial with a parallel observational arm. Centers will decide on a participant by participant basis which arm they should be included in.

Patients meeting the inclusion criteria will be identified in the MDT meeting. The local MDT will decide whether to offer the patient the opportunity to be randomized to one of two treatment arms, or to include them in a parallel observation arm. The trial commences at the point of randomization or enrollment.

### Therapeutic Regimens

#### Surgical Treatment Arm 1 (Staged Procedures)

##### Preoperative Assessment

The following should be assessed before surgery in all trial participants: Assessment of American Society of Anaesthesiologists classification; routine preoperative investigations as recommended by the National Institute for Health and Care Excellence; computed tomography (CT) of the chest or abdomen or pelvis; contrast-enhanced magnetic resonance of the liver and positron emission tomography (PET; either combined with CT or stand-alone) as required to ensure patients meet eligibility criteria, performed up to 28 days before randomization; and a diagnostic laparoscopy may be performed at the discretion of the local unit.

##### Operations

Participants will undergo the first of the two-staged procedures within 2 weeks of enrollment and randomization. The choice of primary first (classic strategy) or liver first (reverse strategy) will be left to the discretion of the recruiting center and addressed in a planned descriptive subgroup analysis. Both resections will be performed within 4 months of enrollment and may be laparoscopic or open and undertaken in different institutions. In this situation, data collection will remain the responsibility of the recruiting (hepatobiliary) unit.

###### Primary Tumor

Standard right, extended right, left or sigmoid colectomy, and anterior resection will be performed under general anesthesia with or without stoma formation dependent upon tumor location and surgeon preference.

###### Liver Metastases

Intraoperative ultrasound evaluation of the liver should be performed to ensure resectability of the liver metastases. The abdomen should be explored to discover extrahepatic abdominal metastases. Suspected extrahepatic abdominal metastases should be confirmed by intraoperative biopsy. The operating surgeon will ultimately decide which procedure is to be performed.

##### Postoperative Procedures

Postoperative care should be provided in line with local hospital procedures.

##### Pathology Specimen

Surgical specimens will be transported as per local hospital protocol and reported in line with the minimum data sets for colorectal cancer and colorectal liver metastases recommended by the Royal College of Pathologists.

Formalin-fixed paraffin embedded (FFPE) blocks from the diagnostic biopsy, primary tumor at the time of resection, and colorectal liver metastasis, again at the time of resection, will be sent to the study team. Snap frozen tissue of colorectal tumor and liver metastasis, as well as normal colorectal mucosa and liver parenchyma, will also be obtained (see section on Translational Research).

##### Time Frames

There are three key time frames within this study. First, participant enrollment and randomization to first surgical procedure should be no more than 2 weeks. In the two-staged group, the time frame between the first and second operation should be no more than 4 months. Finally, if adjuvant chemotherapy is planned (at the discretion of the treating medical oncologist), treatment must be completed within 12 months of randomization.

##### Concomitant Therapy

No concomitant chemotherapy or biological therapy is permitted between the time points of randomization and completion of the surgical strategy, that is, resection at both disease sites. Given this is not logistically possible in the simultaneous arm, interoperative chemotherapy in those undergoing staged procedures would severely bias the trial. Instead, it is anticipated that the participant will proceed to the second procedure as soon as fitness permits. No other anticancer or investigational therapies are permitted while participants are engaged in this study, although participants may be enrolled on other descriptive studies.

##### Participants in the Observational Arm

Participants in the observational arm will undergo exactly the same interventions as those randomized, the only exception being that the staging and order of surgery will be decided by the local MDT rather than being the subject of randomization.

**Inclusion criteria.**

- Histologically confirmed primary colorectal cancer (any T or N stage) in situ treatable with surgical resection
- Minimally symptomatic primary tumor: no radiological evidence of obstruction or perforation and hemoglobin >9 g/dL
- Computed tomography and magnetic resonance imaging and positron emission tomography–confirmed liver-limited metastatic disease (any number and distribution) which in the opinion of the local multidisciplinary team is treatable with surgical resection
- Has completed all necessary neoadjuvant therapy (systemic chemotherapy [any regimen or duration] and/or radiotherapy) for the treatment of stage IV colorectal cancer before entering the trial
- No other known primary tumor or treatment for another tumor type within the 12 months before entering the trial
- Aged 18 years or older with Eastern Cooperative Oncology Group* * performance status 0 to 1 and considered fit to undergo potentially curative treatment (surgery +/− chemotherapy) for their disease, including having recovered from any adverse events associated with neoadjuvant therapy.
- Absence of any psychological, familial, sociological, or geographical condition potentially hampering compliance with the study protocol and follow-up schedule; those conditions should be discussed with the patient before registration in the trial
- Willing to offer written informed consent according to Good Clinical Practice Guideline of the International Conference on Harmonization and national or local regulations
- Patients can only be randomized in this trial once
- Female patients of childbearing age must be willing to utilize contraception

#### Surgical Treatment Arm 2 (Simultaneous Procedure)

The treatment plan for those in the simultaneous group is identical to that described above with the exception of the surgical intervention.

The simultaneous resection of colorectal primary and liver metastases is a pure combination of the procedures previously described with the hepatic resection preceding the colorectal resection. This approach will allow the early reduction of portal pressure, followed by anesthetic manipulation of the mean arterial or central venous pressures to permit a safe colonic resection and anastomosis. Performing the procedure in this sequence also allows completion of the clean part of the operation before the potentially contaminated part.

#### Procedures Applicable to Both Arms

##### Quality Control Procedures

No specific training or accreditation of surgeons will be necessary for this study, given the well-established nature of the surgical techniques under investigation. Resection of liver metastases must be performed by a consultant hepatobiliary surgeon, and resection of the colorectal primary must be performed by a consultant colorectal surgeon. Surgeon participants will be encouraged to continue to submit data to national registries and audits, and specific elements of the pathological dataset, that is, margin positivity and total nodes harvested, will be compared both within and between groups to ensure comparable quality of surgical intervention. Exact details of surgical procedures must be recorded on the case report form.

##### Chemotherapy and Radiotherapy Treatment Plan

The inclusion criteria state that patients must have completed a neoadjuvant regimen in the 4 months before enrollment and randomization. Administered chemotherapy may have been purposefully preoperative or induction chemotherapy with the intent of downstaging for consideration of surgical intervention. In any case, it is anticipated that this regimen will consist of 3 months treatment with either 4 cycles of CAPOX (capecitabine, oxaliplatin) or 6 cycles of FOLFOX (folinic acid, fluorouracil, and oxaliplatin), although the exact regimen will be left to the discretion of the treating medical oncologist. An interval of 4 weeks must be left between completion of chemotherapy and the first operation. For patients with rectal cancer, it is anticipated that neoadjuvant treatment will consist of either short-course radiotherapy or long-course radiotherapy with chemosensitization. The exact details of the neoadjuvant therapy will be at the discretion of the treating oncologist.

Targeted biological therapy (eg, cetuximab, panitumumab or bevacizumab) may also have been administered in the neoadjuvant setting. If so, the standard 6-week interval between administration of bevacizmab and surgical intervention is to be observed. A planned descriptive subgroup analysis will be undertaken to examine for any bias introduced by the uncontrolled use of biologics in the neoadjuvant period.

Following safe completion of surgery at both sites, adjuvant therapy may be administered at the discretion of the treating medical oncologist. This is again anticipated to be a 3-month treatment with either 4 cycles of CAPOX or 6 cycles of FOLFOX.

##### Clinical Evaluation, Laboratory Tests, and Follow-Up

The principle of the follow-up and evaluation strategy is to mirror existing NHS practice so as not to further inconvenience the patient or incur additional trial costs. Data will be collected at the first postoperative review following each procedure (30 [SD 5] days). Reviews at 6 and 12 months following enrollment will be performed with reference to the patient case notes and a telephone interview. The exact same follow-up protocol will be followed for patients in both the randomized and observational arms of the trial.

###### Before Treatment Start

The following data will be collected upon registration:

- Demographics—Age, sex, comorbidities (cardiovascular, respiratory, neurological, endocrine)
- Primary tumor—Location, T stage, N stage, tumor grade on diagnostic biopsy
- Liver metastases—Number, mean size, distribution
- Radiology (CT or contrast magnetic resonance imaging [MRI] or PET)—Confirmation of no extrahepatic metastases
- Biochemistry and hematology—Standard hematology, renal function, liver function, carcinoembryonic antigen (CEA), and carbohydrate antigen 19-9 (CA19-9)
- Details of neoadjuvant chemo(radio)therapy received—number of cycles, agents administered, response evaluation (Response Evaluation Criteria in Solid Tumors v1.1, RECIST v1.1), total radiation dose
- Confirmation of meeting other eligibility criteria
- Baseline EuroQol-5D (EQ5D) and Functional Assessment of Cancer Therapy-Colorectal (FACT-C)
- Planned adjuvant therapy

###### During Treatment

The following data will be collected immediately following each procedure (where relevant):

- Type of colorectal resection performed
- Type of liver resection performed (plus combined local treatment)
- Operation length
- Estimated blood loss
- Transfusion
- Destination of patient following surgery (intensive therapy unit, high-dependency unit, or ward)

###### Follow-Up

####### Thirty (SD 5) Day Postoperative Review (After Every Procedure)

Patients will routinely be followed up face-to-face at 4 to 6 weeks following each surgical intervention. No specific investigations will be required. At this point, the following data will be collected (where applicable):

- Surgical complications as per Clavien-Dindo scale—details and dates of specific complications will be recorded
- Length of critical care stay
- Length of hospital stay
- Total in-hospital blood transfusion
- Pathological dataset for primary tumor—differentiation, margin positivity, nodal yield and positivity, T stage, N stage
- Pathological dataset for colorectal liver metastases—number and distribution of metastases, differentiation
- R0 resection rate
- Biochemistry and hematology—Standard hematology, renal function, liver function, CEA, and CA19-9
- EQ5D and FACT-C (following simultaneous resection and second staged procedure only)

####### Six (SD 1) Months Postrandomization

This review will be conducted with reference to the case notes and a telephone interview. The following data will be collected:

- Surgical complications as per Clavien-Dindo—details and dates of specific complications will be recorded
- Evidence of recurrence or death (ie, disease free survival)
- Whether adjuvant chemotherapy has been commenced, and if so, the number of days following randomization at which this occurred
- Whether the participant has returned to work, and if so, the number of days following randomization at which this occurred

The exact schedule for postoperative radiology and surveillance will not be explicitly specified within the trial protocol; rather, left to the discretion of the treating clinicians and local policy. At each clinical review within the trial, available radiology will be reviewed for evidence of recurrence. To enable an assessment of disease free survival, all patients must have at least two CT scans between the point of randomization and the end of the study.

####### Twelve (SD 1) Months Postrandomization (Protocol End)

Data collection at this point is an exact duplicate of the 6-month time point, with the addition of the EQ5D and FACT-C QoL questionnaires that will be administered by post. In addition, the date of completion of adjuvant chemotherapy (if applicable) will be recorded. One-to-one semistructured interviews will also be conducted at this time point.

####### Postprotocol Data Collection

Data registries will be reviewed annually for 5 years to establish overall survival.

#### Criteria Under Evaluation

Each end point will be individually and specifically evaluated as follows.

##### Primary End Point—Feasibility of Randomization

Feasibility of randomization is to be initially established in a pilot study.

##### Secondary End Points

###### Total (Severe) Complications

Although a number of scoring systems for operative morbidity exist, most trials are reported using the Clavien-Dindo model [17]. Although all complications represent a failure of treatment and impact on the patient to some extent, the investigating team feel that those severe complications (Clavien-Dindo score 3-5), *that is*, requiring reintervention, represent the most significant clinical problem. This end point is defined as the total number of severe complications sustained by the patient within the study period*, which is* 12 months following randomization. It is accepted that this will represent a longer period of postoperative follow-up for those patients in the simultaneous arm of the study, but in each arm, the period of time to identify complications will be more than 6 months. The total number of complications and percentage of patients having complications within the study period will also be reported.

###### Disease Free Survival

Routine clinical follow-up will allow for the radiological evaluation of disease recurrence. Failure in this context is defined as local disease recurrence and/or nodal disease meeting standardized size criteria at the site of the primary tumor, recurrence of metastatic disease in the liver, occurrence of extrahepatic metastatic disease (not limited to the abdomen), and death.

Time to event is defined from the date of randomization until the date of first failure. Patients who remain disease free at the time of analysis will be censored to the date of last visit.

###### Overall Survival

All-cause mortality data will be obtained from national cancer registries outwith the follow-up formally described within the remit of the trial. Time from date of randomization to date of death will be analyzed. Patients still alive will be censored at the date of the last assessment.

###### Achievement of R0 Resection at Both Sites

Achievement of R0 resection must be agreed by the treating surgeon and the histopathologist reviewing the case with reference to both the operative findings and pathological assessment of the surgical specimen.

###### Completion of Planned Adjuvant Therapy

The optimal adjuvant therapy on the assumption of R0 resection at both sites and patient fitness will be documented at the time of randomization. The proportion of patients fulfilling this planned strategy within 12 months of randomization will be analyzed.

###### Time to Commencement of Adjuvant Chemotherapy

This end point will examine the time from the date of randomization to the date of administration of the first dose of adjuvant therapy (if planned).

###### Health Economics

Please see below the details of the planned evaluation under the section Health Economic Evaluation.

###### Quality of Life

Please see below details of the planned evaluation under the section on Quality of Life Assessment.

###### Return to Work

Defined as the number of days from randomization to return to full-time occupational activity after having had resection at both sites, this end point will only apply to those in employment at the time of randomization. No alternative end point will be used for patients not returning to employment.

### Statistical Considerations

#### Statistical Design

##### Sample Size

No formal power calculation has been performed for this draft protocol, which represents a pilot study primarily to establish feasibility of randomization.

##### Randomization and Stratification

This is an unblinded study with online registration and randomization. Three stratification factors will be used: center, colonic (cecum, ascending, transverse, descending, sigmoid) vs rectal primary, and anatomical (+/− local procedures) vs nonanatomical (+/− local procedures) liver resection.

#### Statistical Analysis

With respect to the other outcomes of interest, we will perform comparisons of proportions (total complications, R0 resection at both sites, completing planned adjuvant therapy) using the chi-square test, and time to event end points (disease free survival, overall survival, time to commencement of adjuvant therapy, time to return to work) will be analyzed using Kaplan-Meir methodology and compared between treatment arms using a log-rank test. Please see specific sections on QoL, Health Economics, and Translational Research for the planned analyses of these data.

Three further (descriptive) analyses are also planned to investigate potential confounders between the two arms, specifically, those patients undergoing the “reverse strategy” of staged resections, that is, liver first, differences in the proportion of patients undergoing laparoscopic resection(s), and the use of small molecular inhibitors before enrollment into the study.

### Independent Data Monitoring Committee

An independent data monitoring committee appointed by the Liverpool Clinical Trials Unit will review the progress of the study.

Terms of reference, membership, meeting details, and data to be reviewed will be agreed independently before the start of the study.

### Quality of Life Assessment

#### Rationale

QoL assessment is considered to be an important patient-centered outcome within this study given that

- Operative morbidity is known to impact negatively on patient QoL
- The study compares two different treatment modalities
- The treatment modalities have different intensity and duration

#### Quality of Life Instrument

PROMS are increasingly used in the NHS to report patient experience, benchmark services, and quality assure treatment. These normally consist of a global QoL scale (eg, EQ-5D) and a disease-specific questionnaire. We have selected FACT-C for the evaluation of colorectal cancer specific metrics.

In addition to this, we plan to explore the creation of a new PROM specifically targeting patients presenting with this advanced form of the tumor.

#### Study Design

The two questionnaires (EQ5D and FACT-C) will be administered at three discrete time points: baseline at registration, at the 30-day postoperative review following the synchronous or second staged procedure, and at 12 months following enrollment (ie, end of trial protocol).

The 30-day postoperative data will permit direct comparison of the effects of simultaneous and staged procedures on early postoperative QoL metrics. It is accepted that these will be at different time points from randomization but remains the outcome of primary interest.

The 12-month data will allow for the assessment of late postoperative complications, and being at a fixed time-point from randomization, may prove to be statistically more robust. This will however be confounded by differences between the two groups with respect to adjuvant therapy.

At least 5 (but no more than 10) patients from each arm of the study will be selected at random to participate in one-to-one semistructured interviews to identify patient-reported outcomes of interest that are not adequately described or covered in the EQ5D or FACT-C. Thematic analysis of transcripts will be qualitatively analyzed until saturation of the themes has been achieved.

### Statistical Considerations

Missing data will be replaced by imputation, and all data will be normalized to baseline. QoL analysis will not be formally powered; however, it will be compared at 30 days postoperatively and 12 months postrandomization.

The FACT-C Trial Outcomes Index is a combination of the physical well-being, functional well-being, and colorectal cancer subscale, with an overall score out of 84 and will be the primary tool of interest. A clinically significant difference is considered to be four points [18] and will be assessed for using a two-sample *t* test (or Mann Whitney *U* test if not normally distributed). As well as direct group comparisons, longitudinal data will be examined by regression analysis.

### Health Economic Evaluation

Preliminary work has suggested a cost difference between the two surgical approaches. Data pertaining to hospital length of stay and operative time were extracted from 3 recent clinical studies. In addition, reference costs for two patients from a hepatobiliary unit, one who underwent simultaneous resection and one staged, were obtained. Using cost data from the NHS Patient Level Information Costing System, the variable costs were weighted with respect to the cost drivers along with procedure-specific fixed costs. The weighted cost difference for operative time was £7856.58 in the simultaneous group and £7722.89 in the staged. Length of stay costs were £3921.72 and £6271.61, respectively. Fixed costs (pharmacy, radiology, pathology, and other) totaling £505, critical care costs (£4500), and theatre consumables (£4000) were assumed to be comparable between the two approaches. The total cost for a simultaneous resection therefore was calculated at £20783.30 compared with £22999.50 for staged resections.

The two treatment arms will be analyzed for comparative cost-effectiveness using a decision analytic model taking a UK-centric decision maker’s perspective. Health-related QoL data collected during the trial using EQ-5D, valued using UK population tariffs, will be used to estimate quality-adjusted survival using the standard area under the curve approach. Resource use will focus on hospital costs, including theatre, ward, and critical care, and drug costs, with the costs associated with a longer hospital stay likely to influence the results. The analysis will take the form of a cost-effectiveness analysis, with the outcome measure being the incremental cost-effectiveness ratio (ICER). Uncertainty in the model parameter values will be mitigated with probabilistic sensitivity analysis, which represents parameters using distributions, in this case using bootstrapping with in-trial data.

Subgroup analyses will be undertaken to identify patient subgroups in which the different treatment arms are more or less cost-effective than in the overall patient population.

Scenario analyses will be performed around important model parameters including the source of quality-adjusted life year estimates to compare the generic and disease-specific QoL measures and their implications for the model results. Results will be presented in the form of (1) the base case ICER, (2) ICERs for clinically relevant subgroups, and (3) a cost-effectiveness plane. 

### Translational Research

The study affords the opportunity to obtain a tissue set that will be of interest to the Institute of Translational Medicine at the University of Liverpool. Providing ethical approval can be obtained; samples will also be made available to other research groups on a collaborative basis.

#### Biopsy Strategy

Following delivery of the colorectal specimen, the proximal staple line will be incised and a linear cut made down the antimesenteric border before excising a peripheral sample (5 mm^3^) of tumor using forceps and a scalpel. Following delivery of the liver specimen, an incision will be made through the resected surface to the liver metastasis, with care being taken not to breach the liver capsule; a peripheral sample (5 mm^3^) of tumor will be obtained. Macroscopically normal adjacent colonic mucosa and liver parenchyma (1-2 cm from the tumor) will also obtained. All tissue sampling will be performed in the operating theatre under aseptic conditions followed by immediate stabilization in liquid nitrogen. Routine clinical samples will also be obtained as per local hospital guidelines. Samples will be stored locally in a −80^o^C archiving facility and transported to the University of Liverpool on completion of the study.

#### Outline of Translational Work

All study participants will have received neoadjuvant or preoperative chemotherapy, and the intention of the translational research arm is to identify potential predictive markers of response to this treatment.

The department of Molecular and Clinical Pharmacology at the University of Liverpool has considerable expertise in global proteomic assessment using the technique of isobaric tagging for relative and absolute quantification (iTRAQ). Response evaluation will be established using RECIST v1.1 and also tumor regression grade, with a view to planning the following investigations:

1. iTRAQ assessment of metastatic tumor to identify proteins differentially expressed in responders (partial response, complete response) and nonresponders (stable disease, progressive disease) to the pretrial neoadjuvant chemotherapy
2. iTRAQ assessment of primary tumor to correlate expression of proteins identified in 1
3. Immunohistochemical analysis of FFPE tissue to establish if the proteins identified in 1 and 2 are detectable in the up-front diagnostic biopsy, that is, to evaluate their potential as an up-front predictive biomarker of response.

If funding can be obtained, the same strategy can be implemented with exome sequencing. Beyond this, we have a panel of locally derived potential biomarkers previously identified from smaller clinical studies and *in vitro* work (quinone oxidoreductase 1, nuclear factor-like 2, acetylcholine). These will also be specifically reviewed and reported within the iTRAQ data and assayed for using standard immunoassays. All data generated from the translational arm of the study will undergo descriptive analysis only and will not influence the study design or power.

### Patient Registration or Randomization Procedure

Patients will be recruited in the clinic once the multidisciplinary team ha*s* ensured that all eligibility criteria are met. Once the patient has agreed to participate, they will be registered into either the observational or randomized arms of the trial. If they are entering the randomized trial, they will be immediately randomized by means of an online database hosted on a secure website.

The following details will be collected upon registration:

- Demographics—Age, sex, comorbidities (cardiovascular, respiratory, neurological, endocrine)
- Primary tumor—Location, T stage, N stage, tumor grade on diagnostic biopsy
- Liver metastases—Number, mean size, distribution
- Radiology (CT or contrast MRI or PET)—Confirmation of no extrahepatic metastases
- Details of neoadjuvant chemo(radio)therapy received —number of cycles, agents administered, response evaluation (RECIST v1.1), total radiation dose
- Biochemistry and hematology—Standard hematology, renal function, liver function, CEA, and CA19-9
- Confirmation of meeting other eligibility criteria
- Baseline EQ5D and FACT-C
- Planned adjuvant therapy

Should all eligibility criteria be met, results of randomization will be made instantly available to permit the planning of the next intervention.

### Reporting Adverse Events

#### Definitions

An adverse event (AE) is defined as any untoward medical occurrence or experience in a patient or clinical investigation subject that occurs following surgery or the administration of the trial medication regardless of the dose or causal relationship. This can include any unfavorable and unintended signs or symptoms (such as nausea or chest pain), an abnormal laboratory finding (including blood tests, x-rays or scans), or a disease temporarily associated with the use of the protocol treatment (Good Clinical Practice Guideline of the International Conference on Harmonization, ICH-GCP).

An adverse drug reaction (ADR) is defined as any response to a medical product that is noxious and/or unexpected, related to any dose (ICH-GCP). Response to a medicinal product (used in the above definition) means that a causal relationship between the medicinal product and the AE is at least a reasonable possibility, that is, the relationship cannot be ruled out. An unexpected ADR is any adverse reaction for which the nature or severity is not consistent with the applicable product information (eg, Investigators’ Brochure, ICH-GCP). A serious AE (SAE) is defined as any undesirable experience occurring to a patient, whether or not considered related to the protocol treatment. An SAE that is considered related to the protocol treatment is defined as a serious ADR.

AEs and ADRs that are considered as serious are those that result in death, a life-threatening event, hospitalization, persistent significant disability or incapacity, a congenital anomaly or birth defect, or any other medically important condition (ie, important adverse reactions that are not immediately life-threatening or do not result in death or hospitalization but may jeopardize the patient or may require intervention to prevent one of the other outcomes listed above).

### Reporting Procedure

It is the responsibility of the principal investigator at each research site to report AEs to the trial steering committee. The chief investigator is responsible for onward reporting.

### Ethical Considerations

#### Patient Protection

The responsible investigator will ensure that this study is conducted in agreement with either the Declaration of Helsinki (Tokyo, Venice, Hong Kong, Somerset West, and Edinburgh amendments) or the laws and regulations of the country, whichever provides the greatest protection of the patient. The protocol has been written, and the study will be conducted according to the ICH Harmonized Tripartite Guideline for Good Clinical Practice. The protocol will be approved by the local, regional, or national ethics committees.

#### Subject Identification

Participants will be identified through the hepatobiliary multidisciplinary team meetings and approached for screening if the treating team believes the eligibility criteria are likely to be fulfilled.

#### Informed Consent

All patients will be informed of the aims of the study, the possible AEs, the procedures and possible hazards to which he or she will be exposed, and the mechanism of treatment allocation. They will be informed as to the strict confidentiality of their patient data but that their medical records may be reviewed for trial purposes by authorized individuals other than their treating physician.

It is the responsibility of the individual investigator to translate the enclosed informed consent document. The translated version should be dated and version controlled.

The translated informed consent form is part of the documents to be submitted to the ethics committee for approval. The competent ethics committee for each institution must validate local informed consent documents before the center can join the study. It is the responsibility of the local ethical committee to guarantee that the translation is conforming to the ICH-GCP guidelines.

It will be emphasized that the participation is voluntary and that the patient is allowed to refuse further participation in the protocol whenever he or she wants. This will not prejudice the patient’s subsequent care. Documented informed consent must be obtained for all patients included in the study before they are registered or randomized in the study. This must be done in accordance with the national and local regulatory requirements.

For European Union member states, the informed consent procedure must conform to the ICH guidelines on good clinical practice. This implies that “the written informed consent form should be signed and personally dated by the patient or by the patient’s legally acceptable representative”.

#### Study-Specific Ethical Issues

The trial committee does not anticipate any specific concerns with the conduct of this trial. Both treatment strategies under study are currently in widespread clinical practice, although the criteria for optimum patient selection remain unclear. The follow-up design is aligned to current NHS follow-up procedures to minimize the inconvenience to the patient.

### Patient and Public Involvement

Informal discussions with patients having undergone both simultaneous and staged procedures have suggested a preference for a single combined procedure, although the threshold of morbidity considered acceptable would be difficult to define. This work (alongside colleague and center questionnaires) is currently being undertaken.

The Delphi exercise from the Association of Coloproctology of Great Britain and Ireland has ranked this question as a key area for research [19], which was supported by the Oracle patient consultation exercise held at the Royal College of Surgeons of England [20].

Preliminary proton pump inhibitors work was conducted recently in Liverpool in anticipation of this study to help guide both the scope and construction of the study. Six semistructured one-to-one interviews were conducted by a single investigator (PS) with patients having undergone simultaneous (n=3) or staged (n=3) resections to identify factors that may have influenced their choice of oncosurgical approach. Interviews were transcribed and thematically analyzed to generate codes that were categorized into emergent themes. This thematic analysis identified four key themes: survival or oncological outcome, holistic outcome or return to function, logistical burden, and effect on carers.

## Results

This study is at the protocol development stage only, and as such, no results are available. There is no funding in place for this study and no anticipated start date.

## Discussion

We have presented preliminary work and an outline trial protocol which we anticipate will inform the future development of clinical studies to investigate the management of patients with liver limited stage IV colorectal cancer. We do not believe that the trial we have designed will answer the most significant clinical questions, nor that it is feasible to be delivered within the UK’s NHS at this current time.

## Abbreviations

ADR: adverse drug reaction
AE: adverse event
CA19-9: carbohydrate antigen 19-9
CAPOX: capecitabine, oxaliplatin
CEA: carcinoembryonic antigen
CT: computed tomography
EQ5D: EuroQol-5D
FACT-C: Functional Assessment of Cancer Therapy-Colorectal
FFPE: Formalin-fixed paraffin embedded
FOLFOX: folinic acid, fluorouracil, and oxaliplatin
ICER: incremental cost-effectiveness ratio
ICH-GCP: Good Clinical Practice Guideline of the International Conference on Harmonization
iTRAQ: isobaric tagging for relative and absolute quantification
MDT: multidisciplinary team
MRI: magnetic resonance imaging
NHS: National Health Service
PET: positron emission tomography
PROM: patient-reported outcome measure
QoL: quality of life
RCT: randomized controlled trial
RECIST v1.1: Response Evaluation Criteria in Solid Tumors v1.1
SAE: serious adverse event
